# Supplementary material for: Association of Non-Alcoholic Fatty Liver Disease and Metabolic-Associated Fatty Liver Disease with COVID-19-Related Intensive Care Unit Outcomes: A Systematic Review and Meta-Analysis
Source: Medicina (Kaunas). 2023 Jul 3;59(7):1239. doi: 10.3390/medicina59071239 (PMC10386363; doi:10.3390/medicina59071239)
Supplement: Supplementary file 1 [file medicina-59-01239-s001.zip › medicina-2434223-supplementary.pdf]

Supplementary index

Supplementary Table S1: Search Strategy for studies

No.	Database	Platform	Data coverage	Date of search	Search term	# of results
1	Pubmed.gov	MEDLINE	ALL dates	7/8/2022	((("Non-alcoholic Fatty Liver Disease"[Mesh]) OR "Fatty Liver"[Mesh] OR "Metabolic Syndrome"[Mesh]) AND ("COVID-19"[Mesh] OR "SARS-CoV-2"[Mesh]))<br><br>"Liver stiffness" and "covid"	193<br><br>7
2	Web of science		ALL	7/8/2022	Fatty liver AND COVID<br>Metabolic syndrome and COVID<br>MAFLD AND COVID<br>Liver stiffness and COVID	142<br>110<br>28<br>7
3	Embase		ALL	7/9/2022	('fatty liver':ti,ab,kw OR 'nonalcoholic fatty liver':ti,ab,kw OR 'metabolic fatty liver':ti,ab,kw) AND (covid:ti,ab,kw OR 'coronavirus disease 2019':ti,ab,kw)<br><br>('fibrosis-4 index':ti,ab,kw OR 'nonalcoholic steatohepatitis':ti,ab,kw) AND (covid:ti,ab,kw OR 'coronavirus disease 2019':ti,ab,kw)<br><br>'Liver stiffness':ti,ab,kw AND 'coronavirus disease 2019':ti,ab,kw	247<br><br>35<br><br>5
4	Science direct			7/9/2022	("Fatty liver" OR "nonalcoholic fatty liver disease") and ("covid-19" OR covid OR "Sars co v 2") Only research articles filtered.	308 Since 2019 Only research articles

				and mini reviews
5	Cochrane	7/9/2022	"Fatty liver" AND "Corona Virus "<br><br>metabolic syndrome AND "Corona Virus "<br><br>Liver stiffness and COVID	Trials 0 Reviews 1<br><br>trials 0 reviews 1<br><br>trials 0 reviews 0

Supplementary Table S2: Multivariate analysis on the effect of MAFLD/NAFLD on for ICU Admission, Mechanical ventilation, and Severity COVID-19 outcomes

STUDY	ICU Admission Factors adjusted for in the multivariate analysis	ICU Admission adjusted OR (95% CI)	Mechanical ventilation Factors adjusted for in the multivariate analysis	Mechanical ventilation adjusted OR (95% CI)	COVID-19 Severity Factors adjusted for in the multivariate analysis	Severity adjusted OR (95% CI)
Calapod [36]					FIB-4 index scores	4.89 (1.34-12.3) p = 0.02
Campos [48]						
Chang [49]	sex, age, household income, alcohol consumption, smoking habits, physical activity, hypertension, diabetes mellitus, stroke, coronary artery disease, atrial fibrillation, heart failure, asthma, chronic kidney disease, malignancy, alanine aminotransferase, and total cholesterol	1.92 (1.06–3.50), P = 0.032	sex, age, household income, alcohol consumption, smoking habits, physical activity, hypertension, diabetes mellitus, stroke, coronary artery disease, atrial fibrillation, heart failure, asthma, chronic kidney disease, malignancy, alanine aminotransferase, and total cholesterol	2.38 (1.06–5.35), P = 0.035	sex, age, household income, alcohol consumption, smoking habits, physical activity, hypertension, diabetes mellitus, stroke, coronary artery disease, atrial fibrillation, heart failure, asthma, chronic kidney disease, malignancy, alanine aminotransferase, and total cholesterol	1.77 (1.11–2.82), P = 0.017

Chen [50]	age, sex, race (white, black, or other), recent healthcare exposure (hospitalization or residence at an extended care facility or skilled nursing facility < 90 days before COVID-19 diagnosis), presence of hypertension, and presence of dyslipidemia	1.60 (1.00–2.57)	age, sex, race (white, black, or other), recent healthcare exposure (hospitalization or residence at an extended care facility or skilled nursing facility < 90 days before COVID-19 diagnosis), presence of hypertension, and presence of dyslipidemia	2.51 (1.52–4.16)		
Gao [54]					age and sex	2.61 (1.10–6.23) *p* = 0.030
Hashemi [55]	CLD vs No CLD	1.77 (1.03-3.04), p = 0.04	CLD Vs No CLD	2.08I (1.20-3.6)		
Kim [28]					Adjusted for Another race	3.40 (1.31–8.81); p=.012
Mahamid [56]					Metabolic syndrome and Male	3.32 (3.20–3.561); p=0.001
Mushtaq [35]	Age Obesity	2.36 (1.67–3.33) p<0.000	Age Obesity	2.03 (1.42–2.90) p<0.000*	diabetes mellitus	2.2 (1.5–3.19) p<0.000
Rentsch [57]	FIB-4>3.25	8.40 (2.90-24.28)				
Targher [43]					High FIB-4 score	4.04 (1.22-13.3) p=0.021

Tripon [25]					FIB-4 > 2.67	4.756 (2.68–8.43) p<0.001
Trivedi [26]	HbA1c, CVD, and admission INR and creatinine	0.31 (0.03-3.29) p=0.33	BMI, diabetes, CVD, non-NAFLD chronic liver disease and admission ALT	0.13 (0.02-1.09) p=0.06		
Moctezuma-Velazquez [59]	age, sex, obesity, diabetes, hypertension, oxygen saturation on room air, total lymphocytes, triglyceride levels,	1.71 (0.95-3.06), p = 0.07	age, sex, obesity, diabetes, hypertension, oxygen saturation on room air, total lymphocytes, triglyceride levels,	2.5 (1.20-5.2) p = 0.01		
Yao [62]					age, gender, BMI, hypertension, diabetes	11.057(1.193-102.439), p=0.034
Yoo [29]					hepatic steatosis index (HSI)	1.41 (1.08-1.83)
Zhou [30]					adjusted for age, sex, smoking,	4.07 (1.20-13.79), p=0.02

obesity, diabetes mellitus and hypertension.

Supplementary Table S3. Quality assessment of the studies included based on the NIH (National Institutes of Health) quality appraisal tool for Case-control studies.

Author, Years	Was the research question or objective in this paper clearly stated and appropriate	Was the study population clearly specified and defined	Did the authors include a sample size justification	Were controls selected or recruited from the same or similar population that gave rise to the cases (including the same timeframe)	Were the definitions, inclusion and exclusion criteria, algorithms or processes used to identify or select cases and controls valid, reliable, and implemented consistently across all study participants	Were the cases clearly defined and differentiated from controls	If less than 100 percent of eligible cases and/or controls were selected for the study, were the cases and/or controls randomly selected from those eligible	Was there use of concurrent controls	Were the investigators able to confirm that the exposure/risk occurred prior to the development Of the condition or event that defined a participant as a case	Were the measures of exposure/risk clearly defined, valid, reliable, and implemented consistently across all study participants	Were the assessors of exposure/risk blinded to the case or control status of participants	Were key potential confounding variables measured and adjusted statistically in the analyses, If matching was used, did the investigators account for matching during study analysis
Madan,2022,India	✓	✓	✓	✓	✓	✓	×	×	✓	✓	×	×
Mahamid,2021,Israel	✓	✓	×	✓	✓	✓	CD	×	✓	✓	CD	×
Tripon,2022,France	✓	✓	✓	CD	✓	✓	×	×	✓	✓	×	✓
Trivedi,2021,USA	✓	✓	×	✓	✓	✓	✓	×	✓	✓	×	✓
Vazquez-Medina,2022	✓	✓	×	✓	✓	✓	×	×	✓	✓	×	✓

,Mexico

Supplementary Table S4. Quality assessment of the studies included based on the NIH (National Institutes of Health) quality appraisal tool for Observational Cohort and cross-sectional studies.

Author, Years	Was the research question or objective in this paper clearly stated	Was the study population clearly specified and defined	Was participation rate of eligible person at least 50%	Were all the subjects selected or recruited from the same or similar populations	Was a sample size justification, power description, or variance and effect estimates provided	Were the exposure (s) of interest measured prior to the outcome(s) being measured	Was the timeframe sufficient so that one could reasonably expect to see an association between exposure and outcome	Did the study examine different levels of the exposure as related to the outcome	Were the exposure measures clearly defined valid reliable, and implemented consistently across all study participants	Was the exposure (s) assessed more than once over time	Were the outcome measures clearly defined, valid, reliable, aid implemented consistently across all study participants	Were the outcome assessors blinded to the exposure status of participants	Was loss to follow-up after baseline 20% or less	Were key potential confounding variables measured and adjusted statistically for their impact on the relationship between exposure (s) and outcome (s)
Calapod,2021, Romania	✓	✓	✓	✓	×	✓	✓	✓	✓	×	✓	×	✓	✓
Campos,2021, Spain	✓	✓	✓	✓	×	✓	✓	✓	✓	×	✓	×	✓	×
Chang, 2022,South Korea	✓	✓	✓	✓	×	✓	✓	✓	✓	×	✓	×	×	✓
Chen,2020,USA	✓	✓	✓	✓	×	✓	✓	×	✓	×	✓	×	✓	×
Demir, 2022,Turkey	✓	✓	✓	✓	×	✓	✓	×	✓	×	✓	×	✓	×
Effenberger,2020,Austria	✓	✓	✓	✓	×	✓	✓	×	✓	×	✓	×	✓	×
Forlano,2020,UK	✓	✓	✓	✓	×	✓	✓	✓	✓	×	✓	×	✓	✓

Goa,2021,China	✓	✓	✓	✓	×	✓	✓	✓	✓	×	✓	×	✓	✓
Hashemi,2020,USA	✓	✓	✓	✓	×	✓	✓	×	✓	×	✓	×	×	✓
Huang,2020,China	✓	✓	✓	✓	×	✓	✓	✓	✓	✓	✓	×	✓	✓
Hussain,2021,Pakistan*	✓	✓	✓	✓	×	✓	×	×	✓	×	✓	×	✓	×
Kim,2021,USA	✓	✓	✓	✓	✓	✓	✓	✓	✓	×	✓	×	✓	✓
Mushtaq,2021,Qatar	✓	✓	✓	✓	×	✓	✓	×	✓	×	✓	×	×	✓
Rentsch,2020,USA	✓	✓	✓	✓	×	✓	✓	✓	✓	×	✓	×	×	✓
Targher,2020,China	✓	✓	✓	✓	×	✓	✓	×	✓	×	✓	×	×	✓
Tignanelli,2021,USA	✓	✓	✓	✓	×	✓	✓	×	✓	×	✓	×	×	✓
Velazquez,2022,Mexico	✓	✓	✓	✓	×	✓	✓	×	✓	×	✓	×	✓	×
Vrsaljko,2022,Republic of Croatia	✓	✓	✓	✓	×	✓	✓	✓	✓	×	✓	×	×	×
Wang,2021,China	✓	✓	✓	✓	×	✓	✓	×	✓	×	✓	×	✓	✓
Yao,2021,China	✓	✓	✓	✓	×	✓	✓	×	✓	×	✓	×	✓	✓
Yoo,2021,South Korea	✓	✓	✓	✓	✓	✓	✓	×	✓	×	✓	×	✓	✓
Younossi,2021,USA	✓	✓	✓	✓	×	✓	✓	×	✓	×	✓	×	✓	✓
Zhou,2020,China	✓	✓	✓	✓	✓	✓	✓	✓	✓	×	✓	✓	✓	✓

*Cross-sectional study design

Supplementary Figure S1: Funnel plot and Egger's test showing publication Bias for the need for ICU admission meta-analysis.

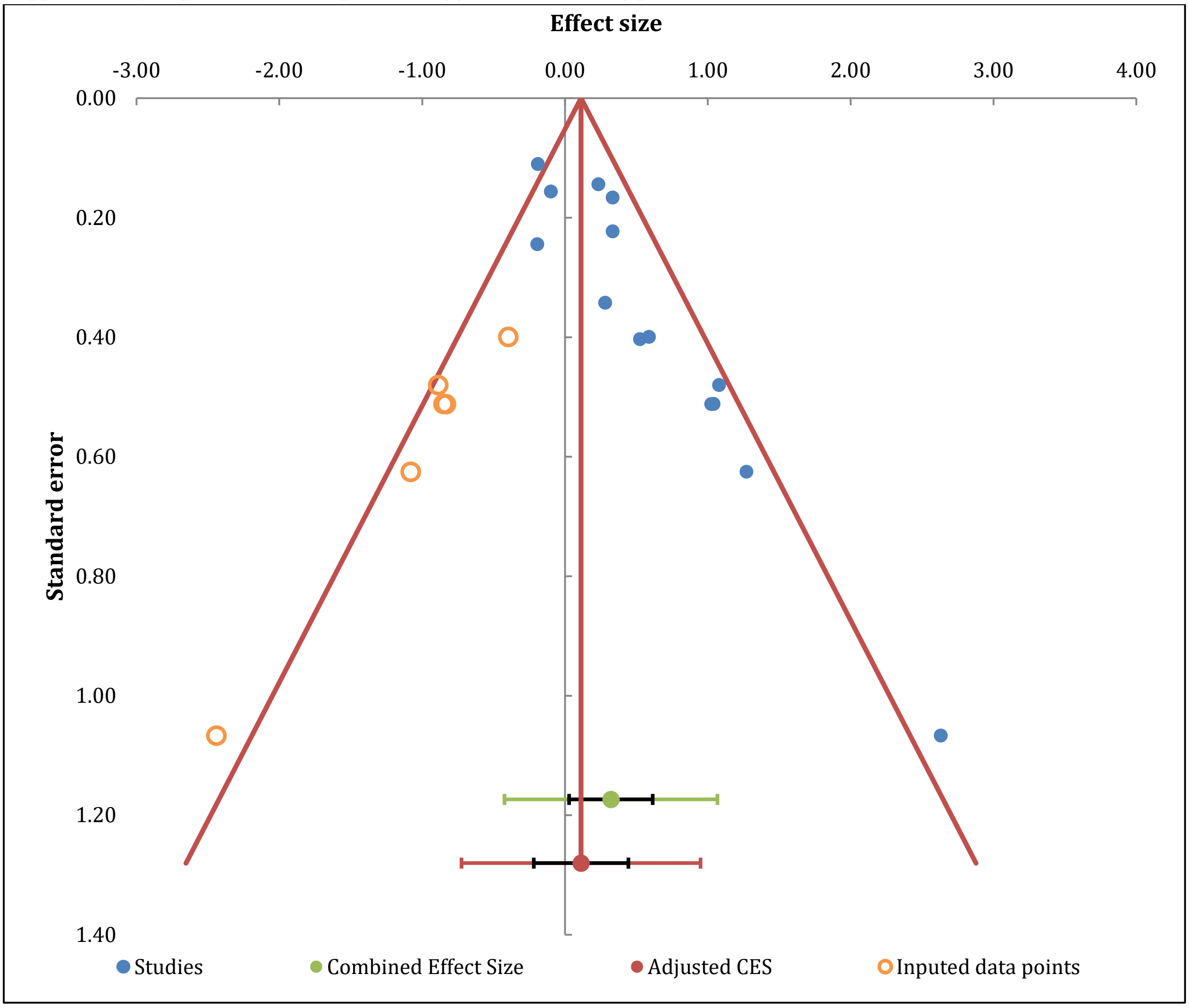

Egger Regression				
	Estimate	SE	CI LL	CI UL
Intercept	1.56	1.20	-0.98	4.10
Slope	-0.82	1.03	-3.01	1.37

t test	1.30
p-value	0.213

Supplementary Figure S2: Funnel plot and Egger's test showing publication Bias for the need for mechanical ventilation meta-analysis.

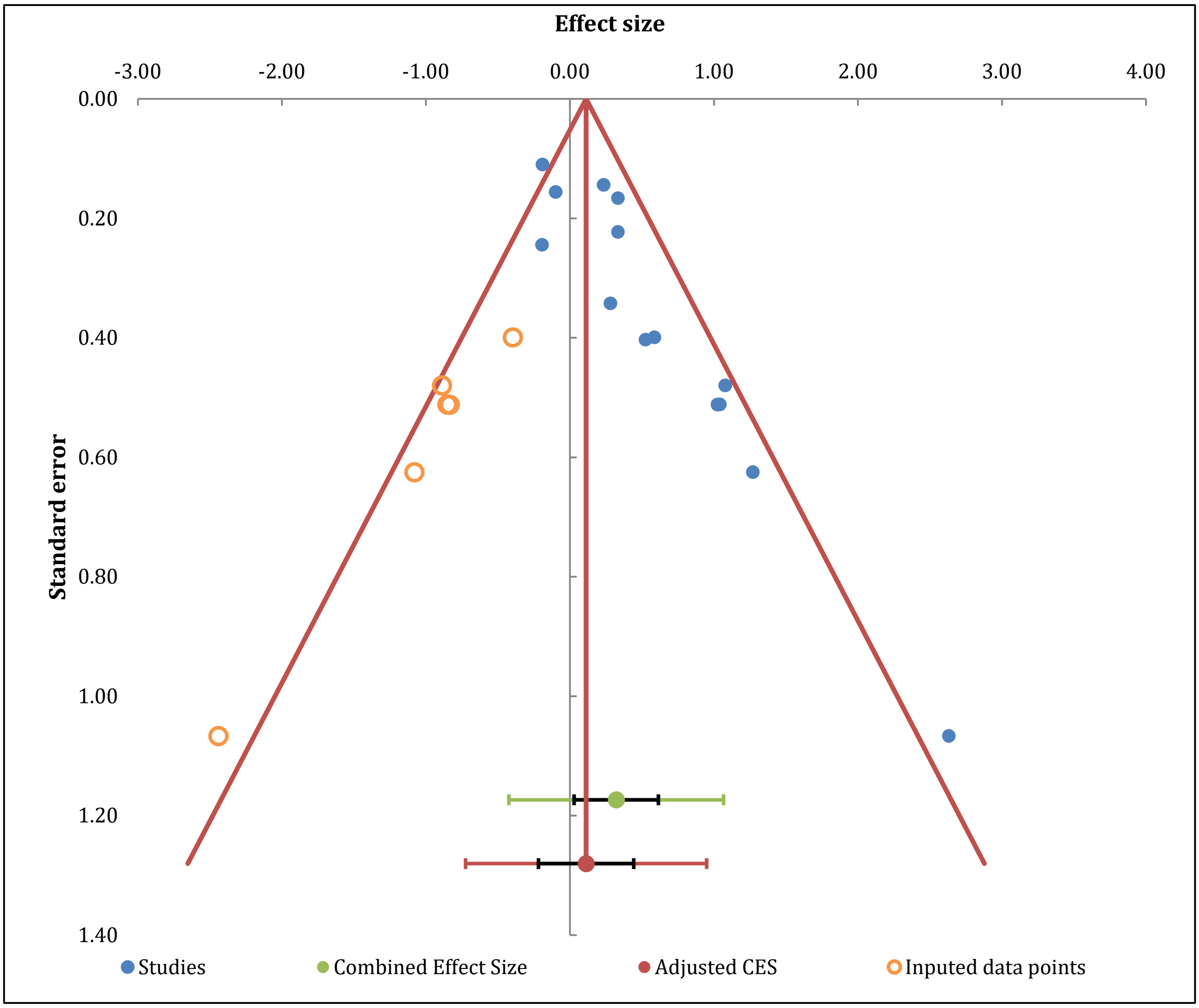

Egger Regression				
	Estimate	SE	CI LL	CI UL
Intercept	-3.29	3.80	-11.57	4.99
Slope	4.01	3.93	-4.55	12.57

t test	-0.87
p-value	0.405

Supplementary Figure S3: Funnel plot and Egger's test showing publication Bias for COVID-19 disease severity meta-analysis.

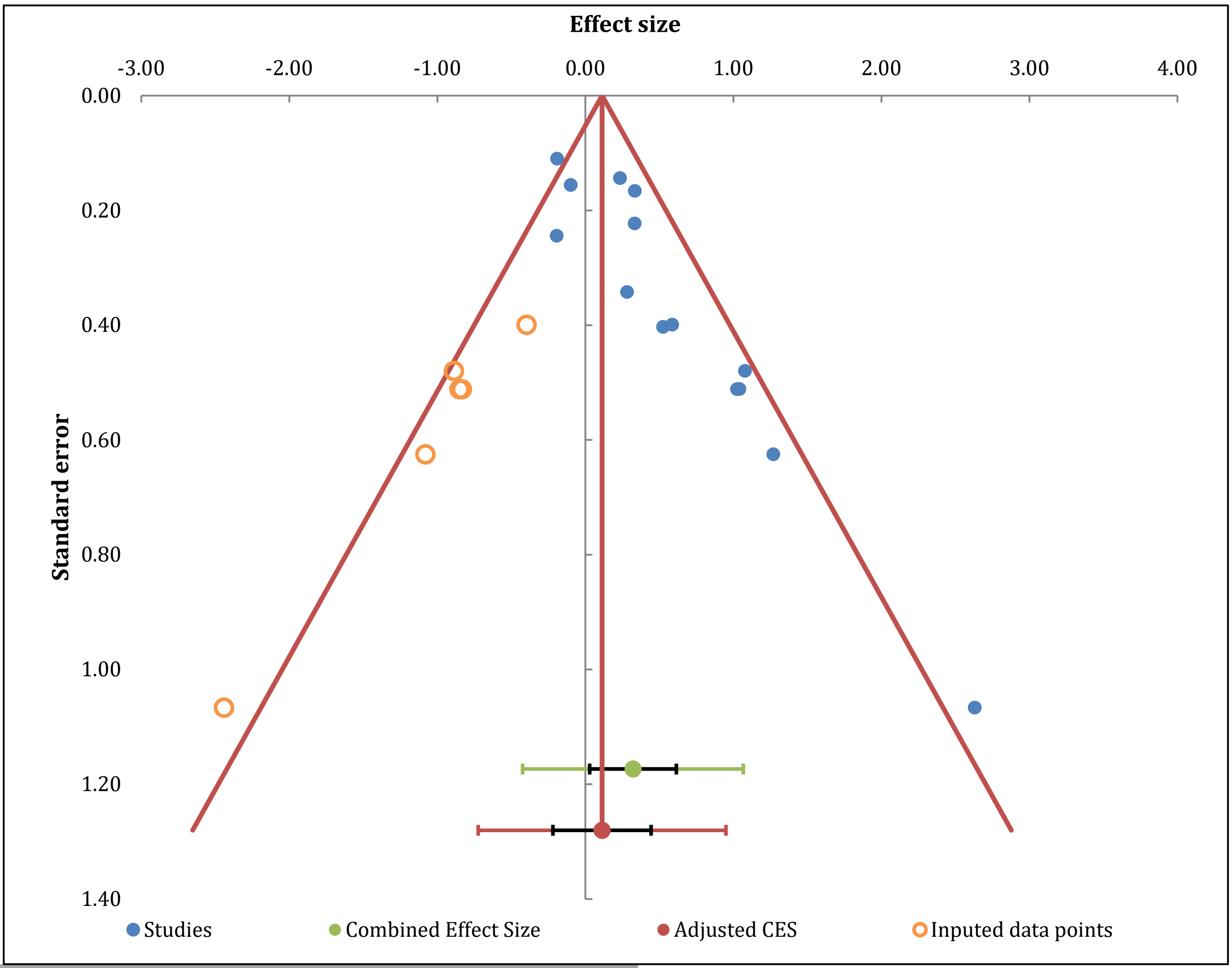

Egger Regression				
	Estimate	SE	CI LL	CI UL
Intercept	3.60	0.49	2.55	4.65
Slope	-1.23	0.22	-1.71	-0.76

t test	7.39
p-value	0.000
